# Spontaneous Coronary Artery Dissection Presenting As ST-Segment Elevation Myocardial Infarction

**DOI:** 10.7759/cureus.25722

**Published:** 2022-06-07

**Authors:** Israa Taha, Timothy Daly, Kashyap Shah, Marc K Antoine, Peter Puleo, Jennifer Axelband, Meredith Birsner

**Affiliations:** 1 Internal Medicine, St. Luke's University Health Network, Bethlehem, USA; 2 Cardiology, St. Luke's University Health Network, Bethlehem, USA; 3 Interventional Cardiology, St. Luke's University Health Network, Bethlehem, USA; 4 Critical Care, St. Luke's University Health Network, Bethlehem, USA; 5 Obstetrics and Gynecology, St. Luke's University Health Network, Bethlehem, USA

**Keywords:** scad in pregnancy, coronary artery dissection, st-elevation myocardial infarction (stemi), myocardial infarction (mi, spontaneous coronary artery dissection (scad)

## Abstract

Spontaneous coronary artery dissection (SCAD) is a non-traumatic spontaneous separation of a coronary wall that can present as acute myocardial infarction. Pregnant females are already at a considerably higher risk of acute myocardial infarction when compared to non-pregnant women of child-bearing age, and dissection explains the majority of these cases. Here, we present a 36-year-old female at 36-weeks gestation who experienced ventricular fibrillation arrest after ST-segment elevation myocardial infarction (STEMI) secondary to spontaneous dissection of the left anterior descending (LAD) coronary artery.

## Introduction

Spontaneous coronary artery dissection (SCAD) separates the coronary arterial wall that is not of traumatic or iatrogenic origin. It is not a common cause of acute myocardial infarction (AMI) in the general population. It has been found in about 0.2% to 4% of acute coronary syndrome (ACS) patients who have undergone invasive angiography [1]. Moreover, coronary artery disease (CAD) incidence in women of child-bearing age is low, and AMI is uncommon [2-3]. However, pregnant women have about three times higher risk of AMI when compared to that non-pregnant women [4]. SCAD represents the most common etiology of pregnancy-associated acute myocardial infarction and has been documented in greater than 40% of the cases on autopsy [4-5].

## Case presentation

A 36-year-old female, gravida, 5 para 2 with a history of 2 prior abortions, hypertriglyceridemia, obesity, tobacco dependence (7.5 pack-year history), and sarcoidosis presented at 36 weeks gestation to the emergency department via emergency medical services after experiencing chest pain at a social event. The pain was sharp, substernal, non-radiating, and started soon after a meal. After she arrived at the emergency department, a seizure developed, and she was given intravenous magnesium sulfate for concerns of eclampsia. Shortly afterward, she became apneic, followed by ventricular fibrillation and cardiac arrest. The obstetrician was contacted given concern for possible emergent hysterectomy, although she quickly had a spontaneous return of circulation after one round of cardiopulmonary resuscitation and single defibrillator shock. She was awake and alert afterward, and her vital signs included a heart rate of 168 beats per minute, a respiratory rate of 26 breaths per minute, a blood pressure of 108/84 mmHg, and an oxygen saturation of 100% on room air. Emergency medicine providers obtained fetal heart tones by ultrasound at 145 beats per minute. She was immediately transferred to the intensive care unit for close monitoring by critical care and an obstetrician. Significant lab results include elevated white blood cell count, liver enzymes, lactic acid, and troponin (Table 1).

**Table 1:** Significant laboratory findings

Test	Result	Reference range
White blood cell count (thousand/uL)	18.13	4.3-10.16
Potassium (mmol/L)	3.2	3.5-4.5
Alanine aminotransferase ALT (U/L)	91	12-78
Aspartate aminotransferase AST (U/L)	79	5-45
Lactic acid (mmol/L)	7.4	0.5-2
Troponin in 1 hour (ng/ml)	4.03	0-0.04
Troponin in 5 hours (ng/ml)	>40	0-0.04

**Figure 1:**
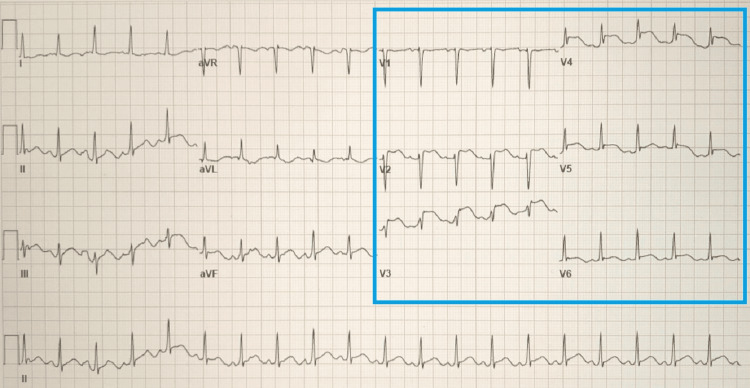
EKG showing ST-segment elevation in leads V2-V5.

**Figure 2:**
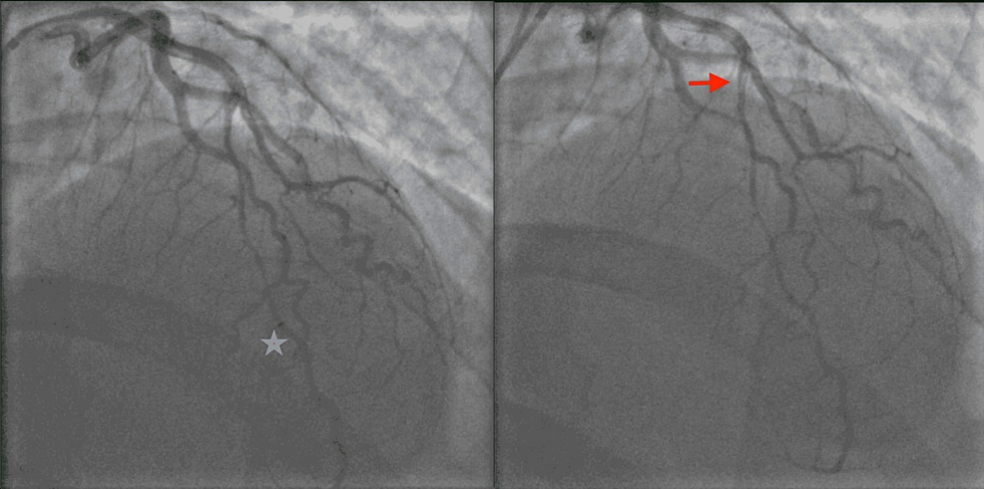
Still left heart catheterization (LHC) image in anterior-posterior (AP) projection with 34-degree anterior-posterior cranial (CRA) of the left coronary system, the red arrow marks the initiation of the dissection plane in the mid-left anterior descending (LAD) and the blue star its approximate termination in the distal segment.

**Figure 3:**
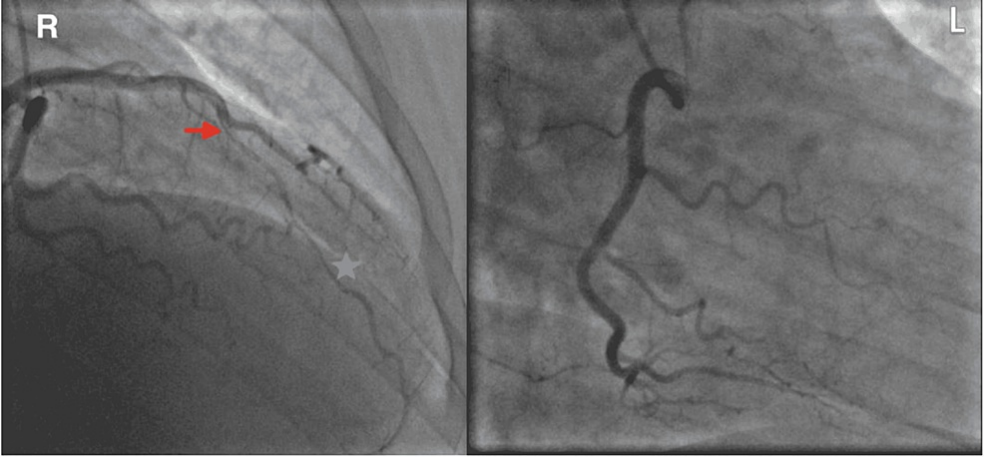
On the right (R): Still LHC image in 30-degree right anterior oblique (RAO) projection with 30-degree caudal (CAU). Again, the beginning and cessation of the LAD dissection plane are delineated by a red arrow and blue star, respectively. Otherwise, the LAD is normal. On the left (L): Straight RAO projection at 30-degree reveals a medium-sized right coronary artery (RCA), free of atherosclerotic disease.

**Figure 4:**
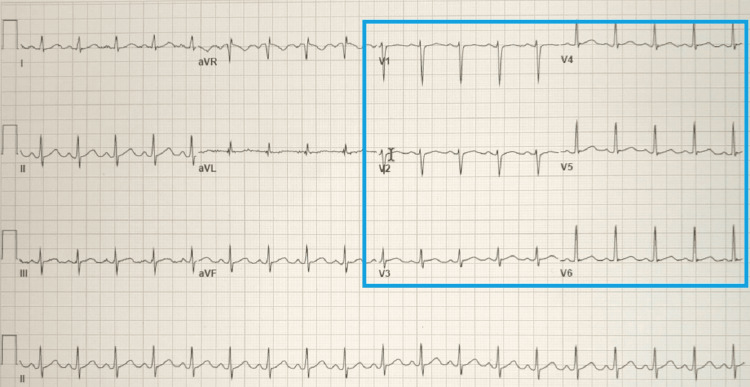
EKG showing resolution of ST elevations in leads V2-V5.

A computed tomography angiography (CT) of the chest was negative for pulmonary embolism and aortic dissection. A CT of the head revealed no acute intracranial abnormality. An electrocardiogram showed ST-segment elevations in leads V2 through V5 concerning a type 1 myocardial infarction (Figure 1). Given STEMI, the patient was given a bolus of 4,000 units of intravenous heparin and immediately taken to the cardiac catheterization lab for coronary angiography. Left heart catheterization (LHC) via right-radial approach revealed a type 1 SCAD extending from the mid vessel segment, immediately past the 1st diagonal (D1) branch, to the distal left anterior descending (LAD) artery with the antegrade flow to the apex; the dissection plane was more appropriately visualized in the anterior-posterior cranial (CRA) and right-anterior-oblique (RAO) caudal (CAU) projections (Figure 2, 3). There was no evidence of atherosclerosis in the LAD or remaining epicardial vessels (Figure 3). Thus no percutaneous coronary intervention was performed. Echocardiography post-procedure revealed an acute decrease in left ventricle ejection fraction (LVEF) at 45% (down from a baseline of 60% about two months prior), elevated filling pressures, and severe hypokinesis of the mid-to-distal anterior, mid-to-distal septal, and apical walls. Electrocardiogram following the procedure revealed resolution of the ST segment elevations (Figure 4). The patient was transferred to another facility within the hospital network in case of the need for cardiothoracic and high-risk OB/GYN care.

Upon arrival, a multidisciplinary conference was held between cardiology, cardiothoracic surgery, critical care medicine, and maternal-fetal medicine. In the setting of an acute MI, the patient was started on metoprolol tartrate, aspirin, and amiodarone infusion for ectopy/ventricular arrhythmia suppression and continued on intravenous heparin. Given the potential for SCAD recurrence and the fetus’ gestational age, a decision was made to proceed with an elective caesarian section after an extensive family discussion. As the patient and significant other were not pursuing further pregnancies, sterilization via tubal ligation was concurrently performed. The patient delivered a healthy infant without any major complications.

**Figure 5:**
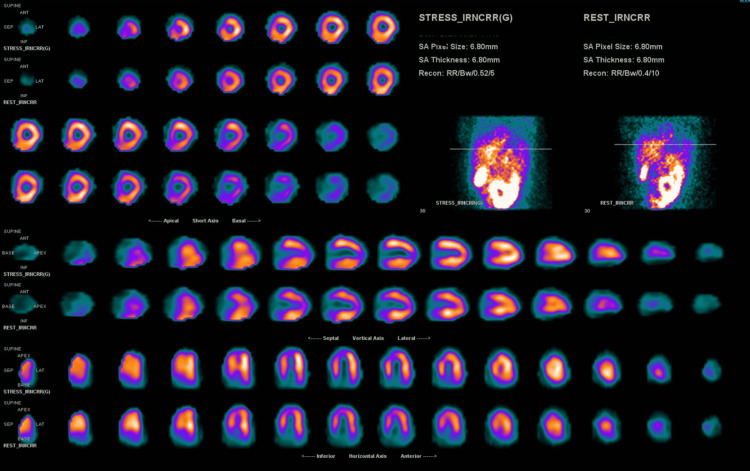
Still image of pharmacological nuclear stress test. There is a small, severe, fixed myocardial perfusion defect of the apical septal wall. There was severely reduced myocardial thickening and motion of the apical wall of the left ventricle; this could not be highlighted as moving frames were not provided.

Post-procedurally, clopidogrel and atorvastatin 40 mg daily were added to her cardiac regimen. An oxytocin infusion was used post-delivery to limit bleeding as heparin infusion was continued peripartum to mitigate the patient’s high risk of coronary thrombus formation. Her course was complicated by postpartum hemorrhage, which required three packed red blood cells transfusion units throughout her hospitalization. After 48 hours of stay in the intensive care unit, the patient was weaned off from all continuous intravenous medications, and her troponin trended significantly downward. She was transferred to a medical-surgical floor and maintained on dual anti-platelet (DAPT) therapy and beta-blockade for one year. Statin therapy was discontinued due to the lack of atherosclerotic disease found on angiography, but lisinopril was added for heart failure guideline-directed medical therapy (GDMT). As a result, she was instructed to refrain from breastfeeding. The patient was discharged home with a wearable defibrillator on the fourth day of her hospitalization. She developed pruritus, attributed to clopidogrel, and was transitioned to ticagrelor. Her LVEF returned to baseline two months after discharge with persisting severe apical septal hypokinesis. Her external defibrillating device was discontinued. A pharmacological nuclear stress test demonstrated a small, fixed perfusion defect with severely reduced thickening in the apical septal wall, consistent with an infarct (Figure 5). She has since maintained routine outpatient cardiology follow-up without recurrence of symptoms or further hospitalizations. She has returned to work and resumed all daily activities with no limitations.

## Discussion

In this case report, we present an interesting case of SCAD in a pregnant female who delivered a healthy newborn without complications during her ST-elevation myocardial infarction (STEMI) hospitalization. SCAD is a life-threatening emergency for both the mother and infant and requires prompt intervention. The mortality rate is as high as 4.2% [6]. The layers of an artery starting from the outermost layer include tunica externa (adventitia) containing collagen and elastic tissue (this includes the vasa vasorum), the tunica media containing smooth muscle, and the tunica intima containing the endothelial cells. The proposed mechanism of non-atherosclerotic SCAD is an intimal tear or bleeding of the vasa vasorum with intermedial hemorrhage, both of which create a false lumen filled with intramural hematoma. The hematoma usually involves the outer two-thirds of the media and triggers an inflammatory reaction in the tunica adventitia [6-7]. The degree of tortuosity of a patient’s vessels can leave certain turning points more susceptible to SCAD [8]. The pressure of the enlarging hematoma can lead to luminal encroachment and subsequent myocardial ischemia and infarction [9]. This is the most common mechanism of acute myocardial infarction in pregnant females, as discussed above. It accounts for nearly a quarter of cases of ACS in women under the age of 50 [10]. In these cases, the dissection may result from increased physiological hemodynamic stresses or hormonal effects weakening the coronary arterial walls [11-12]. This is likely due to excess progesterone during pregnancy, causing the loss of normal corrugation of elastic fibers and degeneration of medial wall collagen resulting in weakening of the arterial wall and leading to arterial dissection. Predisposing conditions for SCAD include fibromuscular dysplasia, postpartum status, multiparity, connective tissue disorders, systemic inflammatory conditions, and hormonal therapy [13-14]. The association of sarcoidosis as a predisposing factor for SCAD is poorly understood. The proposed mechanism involves the breakdown of the tunica media-adventitia layer by inflammatory granulomas of cardiac sarcoidosis predisposing patients to SCAD [15]. Given that our patient was diagnosed with sarcoidosis seven years ago and has been in remission since then, it would be very unusual to have cardiac sarcoidosis presenting with SCAD without any other systemic manifestations.

SCAD patients present with life-threatening ventricular arrhythmia in 4% to 14% of cases and STEMI 25% to 50% of the time [14]. Furthermore, the LAD is the most commonly involved epicardial vessel, making up 40% to 70% of cases [16]. An LHC is the standard diagnostic tool. Three types classify the appearance of SCAD: (1) pathognomonic contrast dye staining of the arterial wall with multiple radiolucent lumens, (2) diffuse long and smooth stenosis that can vary in severity from mild stenosis to complete occlusion, and (3) mimics atherosclerosis with focal or tubular stenosis [17]. As in our case, if coronaries are widely patent, conservative therapy with DAPT, beta-blockade, and statin therapy (if evidence of dyslipidemia or atherosclerosis) is recommended. This approach is favored because revascularization in patients with SCAD is technically challenging and associated with high failure rates or complications [18-19]. The latter includes a progression of vessel wall tear, occlusion of branch vessels after stenting, in-stent restenosis, stent malposition, in-stent thrombosis, and stent migration [20]. However, if the patient continues to have ischemia or begins to have hemodynamic compromise, percutaneous or surgical revascularization should be considered [13-21]. The Canadian SCAD cohort study included 750 SCAD patients, yet only 2% required percutaneous coronary intervention, and 0.3% required CABG [22].

Long-term prognosis has significantly improved, with mortality down to 4.2% [6]. Historically, 38 to 50% of patients succumbed to SCAD [11]. Compared to ACS patients without SCAD, the survival rate is favorable [23]. Routine physical activity is recommended at a moderate level, but a patient should abstain from competitive level training, endurance exercises, exhaustive workouts, and exercises in extreme temperatures. Patients with SCAD should not be discouraged from further pregnancy as recurrence cannot be predicted. Nonetheless, pre-conception counseling, risk factor modification, and multidisciplinary care are central to a successful gestation.

## Conclusions

SCAD is an uncommon cause of acute myocardial infarction (AMI) but should always be considered in a pregnant female with ACS symptoms. Conservative medical management is usually preferred in stable patients because of the friability of the coronary arteries post-dissection. Hemodynamic compromise or persistent symptomatic ischemia are the major indications for invasive intervention. Long-term outcomes are favorable. Further gestations should not be prevented, but close follow-up is required.
